# Exploration of changes in pharmacy students’ perceptions of and attitudes towards professionalism: outcome of a community pharmacy experiential learning programme in Taiwan

**DOI:** 10.1186/s12909-022-03261-6

**Published:** 2022-03-22

**Authors:** Yen-Ming Huang, Hsun-Yu Chan, Ping-Ing Lee, Yun-Wen Tang, Ta-Wei Chiou, Karin C.S. Chen Liu, Yunn-Fang Ho

**Affiliations:** 1 Graduate Institute of Clinical Pharmacy, College of Medicine, National Taiwan University, 100025 Taipei, Taiwan; 2 Department of Industrial Education, National Taiwan Normal University, 106308 Taipei, Taiwan; 3 Department of Pediatrics, National Taiwan University Hospital, National Taiwan University College of Medicine, 100226 Taipei, Taiwan; 4 Department of Pharmacy, National Taiwan University Hospital, College of Medicine, National Taiwan University, 100225 Taipei, Taiwan; 5 School of Pharmacy, College of Medicine, National Taiwan University, 100025 Taipei, Taiwan

**Keywords:** Community, Experiential, Pharmacy, Professionalism

## Abstract

**Background:**

A powerful way to nurture and strengthen professionalism is by accruing practice-based experiences. However, few studies in Taiwan have evaluated the impacts of experiential learning programmes on pharmacy students’ views on professionalism − the core of quality healthcare practices and services. This study aimed to measure changes in perceptions of and attitudes towards professionalism among third-year pharmacy students following an introductory-intermediate experiential learning course.

**Methods:**

A single-group pre- and postcourse comparative study using a self-administered survey was conducted in 2017. Pharmacy students in their third year of a six-year programme were eligible to participate in this study. We used a 28-item questionnaire with a 10-point Likert-type scale to assess students’ professionalism. Among them, 10 items were employed to assess students’ perceived importance of professionalism in pharmacy practice, and another 18 items adapted from the Pharmacy Professionalism Instrument were used to evaluate students’ attitudes towards pharmacy professionalism. An independent t test was performed to compare the differences in students’ anonymous survey responses before and after the course, with an a priori level of statistical significance of 0.05.

**Results:**

Fifty-two pharmacy students participated in the study. They showed significant improvement in three tenets of professionalism, namely, altruism (*p* = 0.035), accountability (*p* = 0.026), and duty (*p* = 0.002), after completing the 5-week experiential course.

**Conclusions:**

Pharmacy students’ attitudes towards professionalism were modifiable by purposely designed experiential learning programme in the community setting. Such experiences may help socialize students with positive attitudes towards altruism, accountability, and duty.

## Introduction

Recent advancements in pharmacy practices and technologies have led to a transformation of pharmacist-patient dynamics worldwide. Instead of staying behind the counter, pharmacists now directly interact with patients or customers, which requires professionalism and professional knowledge and skills [1]. Healthcare personnel who practice a high level of professionalism tend to provide quality patient care and improve health outcomes by building working therapeutic relationships, eventually improving the general public’s perception of and trust in the profession and its members [2]. Over the past three decades, pharmacists in many countries have successfully evolved from a traditional role of merely focusing on dispensing medications to a new role of actively providing direct patient-centred care [3]. To fulfil these extended duties, pharmacy students must cultivate their ability to acquire knowledge and skills and develop professionalism on their way to becoming professionals [4].

Professionalism is defined as the active demonstration of the merits of a profession that focuses on attitudinal and behavioural aspects of being a professional [5]. Although professionalism is both a core competency of the pharmacy profession and an indispensable capstone for pharmacy education, teaching and learning professionalism are often unavoidably neglected due to time constraints and competing curricular priorities [6]. For example, current undergraduate pharmacy curricula in Taiwan emphasize the cultivation of technical competence and academic excellence, such as basic science and clinical knowledge, leaving little room for learners to develop professionalism in the transition from novice to practice-ready professional. The integration of professionalism into curricular design and the development of students’ professionalism have long been overlooked in pharmacy education, partly due to a lack of consensus in defining professionalism and tools for evaluating students’ professionalism [7].

Pharmacists with a good level of professionalism can carry out quality pharmaceutical care, and the approach of learning by example and demonstrating the exhibited behaviours is an effective way to instil pharmacy students with exemplary professionalism [4, 8]. Cultivating professionalism goes beyond the “taught” curricula in pharmacy schools [9]; instead, it must be actively acquired through the process of professional socialization [5]. As Schafheutle et al. suggest, professionalism is most effectively achieved through profession-related activities, such as dispensing sessions, problem-solving activities, and role playing [10]. Student professionalization is positively influenced by interactions with “patient-facing” teaching staff–pharmacist educators who regularly work in community pharmacies [2]. In addition, situated learning theory (SLT) posits that learning is unintentional and embedded within authentic activity, context, and culture [11]. Following SLT, pharmacy students are encouraged to reflect upon and promote their mindfulness of pharmaceutical care by engaging in interactions with patients and collaborating with healthcare team members in community settings. Healthcare novices should be able to cultivate their professionalism by thinking about, understanding, and knowing the healthcare tasks that take place in a plethora of situations (e.g., medication counselling) that are part of the routine practices of a community pharmacist [12]. That is, knowledge needs to be presented in authentic contexts [13]; modern training in pharmacy curricula in the United States introduces experiential education to expose students early on to rich components of pharmacy practices [14, 15]. Pharmacy students have been found to have more confidence and show positive attitudes towards mastering their professionalism when immersed in real-world experiential training [15]. However, such evidence is lacking in Taiwan, even though its pharmacy education has long incorporated experiential learning into the core curriculum. A systematic evaluation of the effectiveness of the curriculum for students’ professionalism is thus necessary to ensure that the curriculum has achieved its intended goal.

A powerful way to nurture and strengthen professionalism is through practice-based experiences. When students participate in an experiential portion of the pharmacy curriculum, assessing professionalism becomes even more necessary [16]. At the School of Pharmacy of National Taiwan University, the target course of the current study, an introductory-intermediate experiential programme in community pharmacy (called *Community Pharmacy Practice Experiences* [CPPE]; see more details below), has been regularly offered for nearly three decades [8]. While this course is required for the six-year PharmD curriculum, no systematic evaluation of students’ professionalism has been performed. The literature supports the need to instil and assess professionalism to support a greater understanding of identity development and to inform teaching, learning, and assessment of professionalism [17]. Collecting quantifiable data is a viable first step in this endeavour since the results will not only inform the future curriculum and instruction but also have implications for career development and advising [18].

Further complicating the evaluation effort is that professionalism varies across cultural contexts and healthcare systems [19]. While a few studies from other countries have described the cultivation of professionalism among pharmacy students [5, 7, 14], limited published work has been performed to investigate pharmacy professionalism in Taiwan [20]. Stipulated by pharmacy law and regulations, pharmacists in Taiwan are fully responsible for completing all the processes involved in prescription dispensing, including repetitive procedures such as counting pills, which cannot be completed by technicians. In contrast, medication therapy management is not regularly carried out in routine practice. In this context of limited cognitive services yet a highly laborious workload for pharmacist practitioners in Taiwan, students may perceive and learn professionalism differently than reported in previous studies [21]. Therefore, this study aimed to evaluate the effect of an introductory-intermediate experiential learning programme on third-year pharmacy students’ views on the professional attitudes required for competent pharmacy practice in community settings. This critical measurement of the changes in perceptions of and attitudes towards professionalism before and after the experiential training programme will help educators tailor course structures and prepare students for a better transition to contemporary pharmacy practice.

## Methods

A single-group pre- and postcourse comparative study using a self-administered survey was conducted to evaluate the impacts of the introductory-intermediate CPPE programme on pharmacy students’ professionalism development. Oral consent was obtained from each eligible adult participant prior to enrolment. Participants were informed of the research purpose and assured of the anonymity of their participation. The collected data were encrypted and password protected according to institutional standards. As this study was meant to help evaluate and improve the course, written consent was not required. The study protocol was approved by the Research Ethics Committee.

### Course overview

**Fig. 1:**
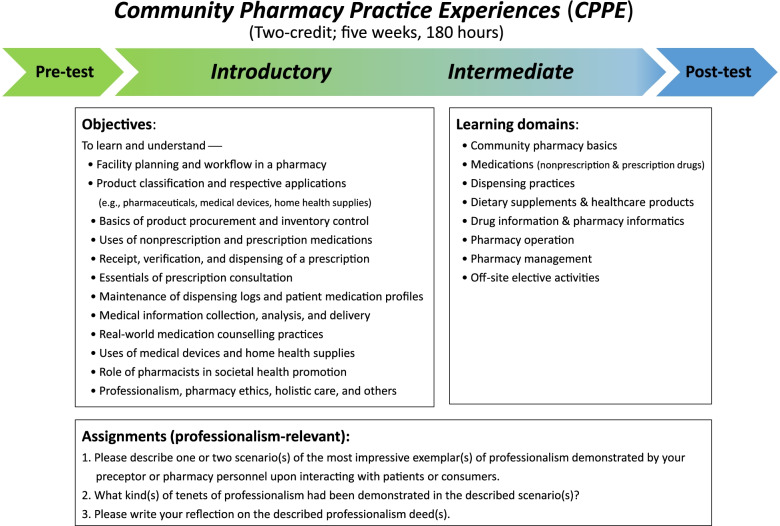
The objectives, learning domains, and assignments in relation to professionalism of the CPPE programme [8, 22]

Since 2009, the School of Pharmacy at the National Taiwan University has implemented the first 6-year Doctor of Pharmacy (PharmD) curriculum in Taiwan in parallel to a 4-year BPharm scheme (1953–2017). The CPPE is an experiential programme for students to observe (*introductory* level; initial weeks) and exercise (*intermediate* level; later weeks) pharmacy service basics, consisting of a 2-credit, 5-week experiential learning course totalling 180 h (Fig. 1) [8, 22]. Third-year (1992–2017; thereafter, changed to fourth-year students due to curriculum restructuring) pharmacy students of the PharmD programme are required to take the CPPE course during summer sessions before entering their fourth year (fifth year after 2017). The majority of students gain their first community pharmacy practice experiences from the rotation, while few might have prior work-study exposures. The study participants had neither introductory hospital rotation nor advanced pharmacy practice experiences until in their fifth and sixth years of pharmacy education, respectively.

All preceptors involving in the CPPE participate voluntarily and hold a Bachelor of Pharmacy degree or higher. The preceptors are required to complete a 16-hour preceptor training workshop that reinforces experiential teaching skills and emphasized the CPPE content to enhance their preceptorship and professionalism [8, 23]. Hence, each preceptor is deemed an exemplary role model from whom pharmacy students can learn during the experiential programme.

The CPPE features four approaches that scaffold students’ understanding, appreciation, and skills for translating abstract knowledge into practice: (1) pharmacist-student apprenticeship, (2) contextualized learning experience in a real community pharmacy, (3) exposure to authentic tasks and patients, and (4) engagement in interprofessional learning [8, 20]. Figure 1 describes the course objectives, learning domains, and professionalism-relevant assignment that are addressed to facilitate students’ professionalism recognition during the CPPE. Pharmacy students shadow pharmacists and learn pharmacists’ routine practices (e.g., become familiarized with the practice environment, dispensing process, and medication counselling) during the first two to three weeks (*Introductory CPPE*). Afterwards, they work with pharmacists (e.g., fill prescriptions, answer patients’ questions, engage in public promotion activities with other students and healthcare team members) under the guidance of a preceptor during the following weeks (*Intermediate CPPE*). Through on-site observation and hands-on exercises, the CPPE provides students with rich opportunities for interacting with mentors, peers, and patients (or customers), which is effective for developing and shaping desirable professional attitudes and behaviours for aspiring future pharmacists.

### Setting and participants

This study was conducted in 2017. A group of third-year pharmacy students were assigned to 20 community pharmacies located in four densely populated urbanized areas in Taiwan, including 7 in Taipei, 11 in New Taipei City, 1 in Taichung, and 1 in Kaohsiung. Pharmacy students with prerequisite knowledge of prepharmacy as well as biomedical and pharmaceutical sciences were eligible to participate in this study. To ensure adequate statistical power, a priori power analysis for a t test with two unpaired groups was conducted using G*Power 3.1, with a presumed effect size (d = 0.80) and α = 0.05 (two-tailed) [24]. As a result, a sample size of 52 participants in total was required to reach a high statistical power (i.e., 0.80) [24, 25].

### Instruments

Students’ professionalism was measured by a 28-item scale developed based on existing literature in pharmacy education while accounting for local healthcare practices [4, 20, 26–30]. Ten tenets of professionalism were identified to describe the full range of professionalism for pharmacy students: altruism, accountability, excellence, communication, honour and integrity, respect for others, duty, ethics, humanism, and teamwork. To ensure the content validity, a preliminary version of the scale was revised by experts in pharmacy education and practice. The experts rated each item on a 5-point Likert-type scale (1 = not fit at all, 5 = perfectly fit) and provided open-ended comments to solicit their insights into the instrument design. A group meeting was conducted to collect the opinions from the experts to discuss the quality of the items. A content validity coefficient based on the Aiken formula was used to evaluate the revised scale after suggestions from the experts were incorporated [31, 32]. The final version of the questionnaire had a content validity coefficient of 0.84, indicating that the scale had an acceptable content validity for measuring professionalism [32, 33].

The above ten tenets were employed to assess students’ perceived importance of professionalism in pharmacy practice [20], and another 18 items adapted from the Pharmacy Professionalism Instrument were used to evaluate students’ attitudes towards pharmacy professionalism [26]. Each item was measured on a 10-point Likert-type scale (1 = not important at all/strongly disagree; 10 = very important/strongly agree) to assess students’ self-rated professionalism. Finally, the participating students provided their demographic background information (i.e., sex and age).

### Data collection and analysis

The students were surveyed before and after the CPPE programme. We used descriptive statistics to summarize the characteristics of the study participants. As the survey was answered anonymously, we were unable to match the pre- and postresponse of each student. Therefore, an independent t test was performed to compare the differences in the students’ survey responses before and after the CPPE. Cronbach’s alpha coefficient was used to test the internal consistency of each scale [34, 35]. All statistical analyses were carried out using SPSS version 26 with an a priori level of statistical significance of 0.05.

## Results

A total of 52 students participated in the CPPE; 52 and 47 of them completed the pre-CPPE and post-CPPE surveys, respectively. Twenty-seven (51.9%) were male students, and the average age was 20.92 years old (SD = 0.97). The Cronbach’s alpha coefficients of the instrument measuring students’ perceived importance of professionalism in pharmacy practice were 0.89 and 0.93 before and after the CPPE, respectively. Additionally, the Cronbach’s alpha coefficients of the instrument measuring students’ attitudes towards pharmacy professionalism were 0.91 and 0.92 before and after the CPPE, respectively. The results indicated that the instruments were acceptable measures of professionalism for pharmacy students [35].

Table 1 shows the results of students’ ratings of the importance of professionalism in pharmacy practice. Of the tenets of professionalism identified, respect for others, communication, and duty were the three highest-rated tenets both before and after the CPPE. None of the ten tenets of professionalism showed a significant improvement between pre- and post-CPPE.

**Table 1:** Students’ perceptions of the importance of professionalism in pharmacy practice [20]

	pre-CPPE (*n* = 52)	post-CPPE (*n* = 47)	Pre-post differences	
	Mean/Median (SD^a^)	Mean/Median (SD^a^)	Mean (SD^a^)	*p* value^b^
Altruism	8.48/9 (1.49)	8.64/10 (1.93)	0.16 (0.34)	0.648
Accountability	8.50/9 (1.82)	8.40/9 (1.86)	-0.10 (0.37)	0.797
Excellence	8.63/9 (1.47)	8.62/9 (1.51)	0.02 (0.30)	0.953
Communication	8.87/9 (1.44)	9.21/10 (1.30)	0.35 (0.28)	0.213
Honour and Integrity	8.44/9 (1.82)	8.87/9 (1.80)	0.43 (0.36)	0.241
Respect for others	9.08/10 (1.20)	9.00/10 (1.57)	-0.08 (0.28)	0.784
Duty	8.85/9 (1.30)	9.09/10 (1.20)	0.24 (0.25)	0.346
Ethics	8.13/8 (1.60)	8.02/8 (1.98)	-0.11 (0.36)	0.314
Humanism	8.35/9 (1.76)	8.53/9 (2.03)	0.19 (0.38)	0.627
Teamwork	7.81/8 (2.22)	8.09/8 (1.61)	0.28 (0.39)	0.483

^a ^*SD* Standard deviation

^b^ The results were examined using independent *t* tests

Table 2 summarizes pre-CPPE and post-CPPE differences in the mean and median scores of students’ attitudes towards pharmacy professionalism. Among the 18 items evaluated, three items showed significant improvement from before to after the CPPE: altruism (i.e., *I do not expect anything in return when I help someone*, *p* = 0.035), accountability (i.e., *I accept the decisions of those in authority*, *p* = 0.026), and duty (i.e., *I attend class/clerkship/work daily as required*, *p* = 0.002).

**Table 2:** Students’ responses to the items on the professionalism instrument [26]

Item		pre-CPPE (*n* = 52)	post-CPPE (*n* = 47)	Pre-post differences	
		Mean/Median (SD^a^)	Mean/Median (SD^a^)	Mean (SD^a^)	*p* value^b^
1.	I do not expect anything in return when I help someone.	6.08/7 (2.57)	7.15/7 (2.39)	1.08 (0.50)	0.035
2.	I attend class/clerkship/work daily as required.	8.58/9 (1.74)	9.47/10 (1.04)	0.89 (0.29)	0.002
3.	If I realize that I will be late, I contact the appropriate individual at the earliest possible time to inform them.	9.00/9 (1.46)	9.30/10 (1.23)	0.30 (0.27)	0.277
4.	If I do not follow through with my responsibilities, I readily accept the consequences.	8.08/8 (1.98)	8.26/8 (1.95)	0.18 (0.40)	0.653
5.	I want to exceed the expectations of others.	8.02/8 (2.01)	8.60/9 (1.64)	0.58 (0.37)	0.123
6.	It is important to produce quality work.	9.00/9 (1.16)	9.17/10 (1.26)	0.17 (0.24)	0.484
7.	I complete my assignments independently and without supervision.	7.71/8 (1.66)	7.98/8 (1.62)	0.27 (0.33)	0.421
8.	I follow through with my responsibilities.	8.60/9 (1.32)	9.02/10 (1.36)	0.43 (0.27)	0.118
9.	I am committed to helping others.	8.19/8 (1.81)	8.36/9 (1.96)	0.17 (0.38)	0.655
10.	I would take a job where I felt I was needed and could make a difference even if it paid less than other positions.	6.17/7 (2.45)	6.40/7 (2.83)	0.23 (0.53)	0.662
11.	It is wrong to cheat to achieve higher rewards.	8.77/10 (2.12)	9.23/10 (1.37)	0.47 (0.36)	0.195
12.	I would report a medication error even if no one else was aware of the mistake.	8.06/8 (1.49)	8.30/8 (1.63)	0.24 (0.31)	0.445
13.	I am open to accept constructive criticism.	8.38/8 (1.47)	8.40/8 (1.42)	-0.02 (0.29)	0.946
14.	I treat all patients with the same respect, regardless of perceived social standing or ability to pay.	8.63/9 (1.70)	8.96/9 (1.10)	0.32 (0.29)	0.262
15.	I address others using appropriate names and titles.	8.67/9 (1.45)	8.85/9 (1.22)	0.18 (0.27)	0.512
16.	I am diplomatic when expressing ideas and options.	7.94/8 (1.59)	8.23/8 (1.59)	0.29 (0.32)	0.364
17.	I accept the decisions of those in authority.	7.33/7 (2.12)	8.17/8 (1.51)	0.84 (0.37)	0.026
18.	I am respectful to individuals who have different backgrounds than mine.	8.87/9 (1.59)	8.98/9 (1.38)	0.11 (0.30)	0.706

*Altruism* is identified by items 1, 9, and 16; *Duty* by items 2 and 3; *Excellence* by items 4–8; *Honour and integrity* by items 10 and 11; *Accountability* by items 12 and 17; *Respect for others* by items 13–15, and 18

^a ^*SD* Standard deviation

^b^ The results were examined using independent *t* tests

## Discussion

This study evaluated students’ perceptions of and attitudes towards pharmacy professionalism over an introductory-intermediate experiential learning programme in community pharmacies. Professionalism, as a tacit integration of knowledge, attitudes, and behaviours, is best learnt through situated learning and real-world experience, which encourages self-reflection and promotes mindfulness [36]. In this study, students had the highest post-CPPE mean score in communication, which lays the foundation for day-to-day workflow in community pharmacies and is frequently documented in the literature [4, 37]. It is plausible that the CPPE contextualizes professional communication skills in a real-world environment, and such a course design can effectively socialize pharmacy students regarding listening and expressing in the stimulating milieu of peer encouragement, preceptor demonstration, patients’ responses, and societal expectations [14]. Community pharmacists regularly perform patient counselling, cooperate with other pharmacists, and negotiate with pharmaceutical companies [38, 39]. Due to their impressionable nature and developing minds, pharmacy students will imperceptibly learn from and imitate their preceptors’ exemplary deeds. In particular, positive role modelling is a powerful means of improving professionalism [14]. The development of professionalism is influenced by role models (e.g., faculty, preceptors, residents, and peer students), the learning environment, and a clear understanding of institutional expectations [40]. These diverse real-life experiences gained from the CPPE may explain why the pharmacy students showed great changes in their perceived importance of communication and teamwork in pharmacy professionalism.

The scores of most of the 18 items assessing students’ attitudes towards pharmacy professionalism improved after the CPPE, but significant differences in the mean scores of students’ attitudes towards pharmacy professionalism were found for only three items. One of the reasons may be the existence of a ceiling effect; for example, items with a high score before the CPPE may not show much improvement afterwards.

The students’ responses showed significant improvements in helping others without expecting rewards (altruism), attending work regularly as required (duty), and accepting the decisions of those in authority (accountability). These improvements regarding attitudinal professionalism may be associated with peer encouragement, the practice environment, and patient and societal expectations [16]. The impact of professional socialization is strong in the practice environment, where pharmacy students are able to learn professional norms through hands-on practice [5]. For example, the CPPE can truly serve as an arena where students practice the tenets of professionalism, which they learn through various learning moments, including direct-service experiences, shadowing of pharmacist practice, and interactions with other healthcare agencies, healthcare providers, and their peers. To be a good team player, students need to be respectful of others’ opinions and responsible for the tasks assigned by preceptors or pharmacists to ensure alignment in the process of patient care.

When they are exposed to experiential training in community pharmacies, students have the opportunity to interact with patients, shadow pharmacists, and work with students from other schools. Such a training environment in community settings may inspire pharmacy students to learn teamwork, collaboration, and workflow in real practice, which includes elements of pharmacy professionalism (i.e., altruism). For example, a teachable moment may occur when a student observes that a pharmacist adeptly fulfils the needs of a difficult patient. A previous study indicated that altruism is one of the tenets of professionalism that students can learn from the community setting, partially due to stronger bonds to the community than to the hospital [41]. Further research is suggested to explore plausible mechanisms through which professionalism grows over time in pharmacy students.

This study illustrates the first step in understanding students’ possible changes in professionalism through use of a valid instrument. Due to the nature of the study design, some limitations exist. First, the research findings are not readily generalizable, as the study was conducted at one pharmacy school; thus, the findings should be regarded preliminary. Therefore, future research should recruit more diverse participants and design comparison groups to determine if the findings are upheld in different cohorts. Second, professionalism and exhibition thereof may change when in a different working environment. Since the statements of the scale used in the study lacked specific context, students may provide different responses in another context and the findings may not be extrapolated to other settings. However, the scale used in the study could be the first step to measure professionalism-related attitudes for pharmacy students. Further studies should provide specific contexts and validate the scale under different contexts for better inferences. Third, this study measured changes in students’ professionalism over a brief timeframe of 5 weeks. We do not know if these changes would persist over time or if explicit changes in certain tenets of professionalism require more time to be nurtured. Subsequent studies should follow up changes in professionalism for a longer timeframe to explore temporal effects on students’ retention of professionalism. Of note, the unprecedented COVID-19 pandemic has had far-reaching impacts on health care, socioeconomic, and educational systems worldwide [42, 43]. The public health roles of pharmacists have been recognized, yet at the expense of greater workload and substantial stress for frontline professionals [44–46]. Future studies should examine changes in professionalism perceived by students since COVID. Fourth, the score of the professionalism scale may have been inflated due to the self-report nature of the scale and may not have reflected the students’ actual professionalism due to the Hawthorne effect, self-improvement bias, and repeat testing bias. Overall, the changes in measured responses might only suggest that the intervention changed the way students responded to the questions, not necessarily reflecting actual improvements in attitudes. Unexpectedly, the perception of the importance of ethics decreased, although not significantly. Further studies could consider using a mixed methods approach to investigate the reasons and mechanisms of changes in specific dimensions of students’ professionalism over time due to the CPPE [47]. Such a design would help pinpoint critical factors that influence students’ professionalism and aid pharmacy educators when tailoring pharmacy curricula to community settings to cultivate holistic professionalism for pharmacy students outside the classroom. The findings will also ease students’ transition to different practice environments in the future.

## Conclusions

Pharmacy students’ attitudes towards professionalism were modifiable by purposely designed experiential learning programme in the community setting. Such experiences may help socialize pharmacy students to have better professional attitudes and practice in community pharmacy settings.

## Data Availability

The study materials and the details of all analyses are available from the corresponding author upon reasonable request.

## Abbreviations

CPPE: Community Pharmacy Practice Experiences
SD: standard deviation

## References

[1] Mossialos E, Courtin E, Naci H, Benrimoj S, Bouvy M, Farris K (2015). From “retailers” to health care providers: transforming the role of community pharmacists in chronic disease management. Health Policy.

[2] Mylrea MF, Gupta TS, Glass BD (2015). Professionalization in pharmacy education as a matter of identity. Am J Pharm Educ.

[3] Wen MF, Lin SJ, Yang YH, Huang YM, Wang HP, Chen CS (2007). Effects of a national medication education program in Taiwan to change the public’s perceptions of the roles and functions of pharmacists. Patient Educ Couns.

[4] Hammer DP, Berger BA, Beardsley RS, Easton MR (2003). Student professionalism. Am J Pharm Educ.

[5] Hill WT. White paper on pharmacy student professionalism: what we as pharmacists believe our profession to be determines what it is. J Am Pharm Assoc (1996). 2000;40(1):96–102. 10665257

[6] Howell BA, Kristal RB, Whitmire LR, Gentry M, Rabin TL, Rosenbaum J (2019). A systematic review of advocacy curricula in graduate medical education. J Gen Intern Med.

[7] Wilson S, Tordoff A, Beckett G. Pharmacy professionalism: a systematic analysis of contemporary literature (1998–2009). Pharm Educ. 2010;10:27–31.

[8] Tang YW, Huang YM, Lo YH, Liu KCSC, Chen LJ, Ho YF (2016). Learning by doing at community pharmacies: objectives and outcomes. J Med Education.

[9] Schafheutle EI, Hassell K, Ashcroft DM, Harrison S (2013). Organizational philosophy as a new perspective on understanding the learning of professionalism. Am J Pharm Educ.

[10] Schafheutle EI, Hassell K, Ashcroft DM, Hall J, Harrison S (2012). How do pharmacy students learn professionalism?. Int J Pharm Pract.

[11] Lave J, Wenger E. Situated learning: legitimate peripheral participation. New York: Cambridge University Press; 1991.

[12] Sørensen EW, Haugbølle LS, Herborg H, Tomsen DV. Improving situated learning in pharmacy internship. Pharm Educ. 2005;5(4):223–33.

[13] Lave J, Illeris K (2009). The practice of learning. Contemporary theories of learning.

[14] Hammer D (2006). Improving student professionalism during experiential learning. Am J Pharm Educ.

[15] O’Sullivan TA, Sy E (2017). A qualitative study designed to build an experiential education curriculum for practice-ready community pharmacy-bound students. Am J Pharm Educ.

[16] Shtaynberg J, Rivkin A, Shah B, Rush S (2013). A quantitative professionalism policy in a community pharmacy introductory pharmacy practice experience. Am J Pharm Educ.

[17] Schwartz A, Ruble M, Sellers KC, Rodriguez-Snapp N, Hill A, Tipparaju S (2018). Incorporation of professionalism expectations and evaluative processes within a college of pharmacy. Am J Pharm Educ.

[18] Dubbai H, Adelstein BA, Taylor S, Shulruf B (2019). Definition of professionalism and tools for assessing professionalism in pharmacy practice: a systemic review. J Educ Eval Health Prof.

[19] Bumgarner GW, Spies AR, Asbill CS, Prince VT (2007). Using the humanities to strengthen the concept of professionalism among first-professional year pharmacy students. Am J Pharm Educ.

[20] Ho YF, Hsieh LL, Lee PI, Chiou TW, Tang YW, Huang YM (2018). Professionalism and establishment of exemplars in pharmacy practice. Formosan J Med.

[21] Kawaguchi-Suzuki M, Law MG, Prisco J, Head K, Fu L, Yumoto T (2019). Cultural sensitivity and global pharmacy engagement in Asia: China, Japan, South Korea, and Taiwan. Am J Pharm Educ.

[22] Tang YW, Kung FL, Liu KCSC, Hsieh LL, Hale KM, Ho YF. Determinants of curricular effectiveness for a community pharmacy experiential course. J Med Education. 2014(3);18:124–134.

[23] Ho YF, Wang HP, Liu KCSC, Tang YW, Wen YH, Wang TC (2017). Preceptor training workshops facilitate quality experiential programs in pharmaceutical primary care. J Med Education.

[24] Faul F, Erdfelder E, Buchner A, Lang AG (2009). Statistical power analyses using G* Power 3.1: Tests for correlation and regression analyses. Behav Res Methods.

[25] Fritz CO, Morris PE, Richler JJ (2012). Effect size estimates: current use, calculations, and interpretation. J Exp Psycholo Gen.

[26] Chisholm MA, Cobb H, Duke L, McDuffie C, Kennedy WK (2006). Development of an instrument to measure professionalism. Am J Pharm Educ.

[27] Poirier TI, Gupchup GV (2010). Assessment of pharmacy student professionalism across a curriculum. Am J Pharm Educ.

[28] Zlatic T (2010). Reaffirming the human nature of professionalism. Clinical faculty survival guide.

[29] Ho MJ, Yu KH, Hirsh D, Huang TS, Yang PC (2011). Does one size fit all? Building a framework for medical professionalism. Acad Med.

[30] Pan H, Norris JL, Liang YS, Li JN, Ho MJ (2013). Building a professionalism framework for healthcare providers in China: a nominal group technique study. Med Teach.

[31] Aiken LR (1980). Content validity and reliability of single items or questionnaires. Educ Psycholo Meas.

[32] Aiken LR (1985). Three coefficients for analyzing the reliability and validity of ratings. Educ Psychol Meas.

[33] Wynd CA, Schmidt B, Schaefer MA (2003). Two quantitative approaches for estimating content validity. West J Nurs Res.

[34] Crocker L, Algina J (1986). Introduction to classical and modern test theory. Rinehart and Winston, 6277 Sea Harbor Drive.

[35] Tavakol M, Dennick R (2011). Making sense of Cronbach’s alpha. Int J Med Educ.

[36] Cruess RL (2006). Teaching professionalism: theory, principles, and practices. Clin Orthop Relat Res.

[37] Boyle CJ, Beardsley RS, Morgan JA, Rodriguez de Bittner M (2007). Professionalism: a determining factor in experiential learning. Am J Pharm Educ.

[38] Huang YM, Chen LJ, Hsieh LL, Chan HY, Chen-Liu KC, Ho YF (2022). Evaluation of use, comprehensibility and clarity of over‐the‐counter medicine labels: consumers’ perspectives and needs in Taiwan. Health Soc Care Community.

[39] Huang YM, Wang YH, Chan HY, Chen LJ, Hsieh LL, Lee PI (2021). Engaging consumers in wise use of over-the-counter medications in Taiwan: development and evaluation of a structured medication counseling approach. Patient Educ Couns.

[40] Roth MT, Zlatic TD, American College of Clinical Pharmacy (2009). Development of student professionalism. Pharmacotherapy.

[41] Javadi M, Asghari F, Salari P (2011). Assessment of professionalism in Iranian pharmacists. J Med Ethics Hist Med.

[42] Ho YF, Hsieh LL, Chao WK, Huang YC (2020). Orientation to Community Pharmacy by online education amid the COVID-19 pandemic: teaching and learning reflections. Journal of Asian Association of Schools of Pharmacy.

[43] Louiselle K, Elson EC, Oschman A, Duehlmeyer S (2020). Impact of COVID-19 pandemic on pharmacy learners and preceptors. Am J Health-Syst Pharm.

[44] Bhamra SK, Parmar J, Heinrich M (2021). Impact of the coronavirus pandemic (COVID-19) on the professional practice and personal well-being of community pharmacy teams in the UK. Int J Pharm Pract.

[45] Jovičić-Bata J, Pavlović N, Milošević N, Gavarić N, Goločorbin-Kon S, Todorović N (2021). Coping with the burden of the COVID-19 pandemic: a cross-sectional study of community pharmacists from Serbia. BMC Health Serv Res.

[46] Aruru M, Truong HA, Clark S (2021). Pharmacy Emergency Preparedness and Response (PEPR): a proposed framework for expanding pharmacy professionals’ roles and contributions to emergency preparedness and response during the COVID-19 pandemic and beyond. Res Social Adm Pharm.

[47] Rao D, Shiyanbola OO (2022). Best practices for conducting and writing mixed methods research in social pharmacy. Res Social Adm Pharm.

